# Analysis of Amino Acid Variation in the P2 Domain of the GII-4 Norovirus VP1 Protein Reveals Putative Variant-Specific Epitopes

**DOI:** 10.1371/journal.pone.0001485

**Published:** 2008-01-23

**Authors:** David J. Allen, Jim J. Gray, Chris I. Gallimore, Jacqueline Xerry, Miren Iturriza-Gómara

**Affiliations:** 1 Enteric Virus Unit, Virus Reference Department, Centre For Infections, Health Protection Agency, London, United Kingdom; 2 Department of Infectious and Tropical Diseases, London School of Hygiene and Tropical Medicine, London, United Kingdom; The Rockefeller University, United States of America

## Abstract

**Background:**

Human noroviruses are a highly diverse group of viruses classified into three of the five currently recognised *Norovirus* genogroups, and contain numerous genotypes or genetic clusters. Noroviruses are the major aetiological agent of endemic gastroenteritis in all age groups, as well as the cause of periodic epidemic gastroenteritis. The noroviruses most commonly associated with outbreaks of gastroenteritis are genogroup II genotype 4 (GII-4) strains. The relationship between genotypes of noroviruses with their phenotypes and antigenic profile remains poorly understood through an inability to culture these viruses and the lack of a suitable animal model.

**Methodology/Principal Findings:**

Here we describe a study of the diversity of amino acid sequences of the highly variable P2 region in the major capsid protein, VP1, of the GII-4 human noroviruses strains using sequence analysis and homology modelling techniques.

**Conclusions/Significance:**

Our data identifies two sites in this region, which show significant amino acid substitutions associated with the appearance of variant strains responsible for epidemics with major public health impact. Homology modelling studies revealed the exposed nature of these sites on the capsid surface, providing supportive structural data that these two sites are likely to be associated with putative variant-specific epitopes. Furthermore, the patterns in the evolution of these viruses at these sites suggests that noroviruses follow a neutral network pattern of evolution.

## Introduction

Noroviruses are the predominant aetiological agent of outbreaks of infectious gastroenteritis, and are typically associated with winter seasonality. Outbreaks are mainly reported in hospitals and elderly care homes, but have also been associated with other semi-closed environments such as schools, restaurants, cruise ships and hotels [1], [2]. Disease caused by these viruses is usually mild and self-limiting, which generally subsides 24–48 hours after onset. However, due to their short incubation time, low infectious dose, efficient transmission and the short duration of protective immunity, noroviruses impact significantly on public health, causing significant morbidity in the population and impose a significant economic burden on national health services [1], [3],

Noroviruses are small non-enveloped viruses with a non-segmented single-stranded, positive sense, RNA genome, which encodes the viral structural and non-structural proteins in three open reading frames (ORF). ORF1 encodes a polyprotein that is post-translationally cleaved by the viral protease into the non-structural proteins including a VPg-like protein, viral protease and an RNA-dependent RNA polymerase (RdRp). The second open reading frame, ORF2, encodes the major structural protein VP1, which has been shown to be organised into distinct domains [4] including a conserved shell (S) domain which forms the contiguous shell of the virus, and a protruding (P) domain which extends away from the capsid. This protruding domain is organised into a highly variable P2 region which is flanked on either side by more conserved P1-1 and P1-2 domains. The P2 domain is the hypervariable region of the capsid and has been suggested that the receptor binding function and antigenic properties of the capsid are localised in this region [5], [6]. The ORF3 encodes a small basic protein believed to be involved in the assembly of progeny particles [7].

There is huge genetic diversity among the human noroviruses, and the populations of viruses co-circulating change between each norovirus season and within a season [8], [9]. The high degree of variation observed among the noroviruses is caused by two mechanisms: mutations as a result of the lack of proof reading associated with RNA replication and by homologous recombination, principally at the junction between the ORF1 and ORF2, and also within the ORF2 [10]–[15].

The classification of noroviruses is based on the sequence analysis of the gene encoding the capsid, however, it has been shown that analysis of conserved fragments of the ORF1 and ORF2 also provides reliable classification, and for molecular epidemiology purposes, this is a widely used strategy [8], [16]. The majority of human noroviruses are divided between two genogroups (GI and GII) and at least 15 genetic clusters within these [17]. The most frequently detected noroviruses associated with outbreaks belong to genogroup II, and are of genotype 4 (GII-4) [8].

In 2002, a GII-4 norovirus genetic variant spread within populations around the world causing large epidemics throughout the year and was characterised by an atypically large number of outbreaks during the summer and autumn months [18], [19]. It is unknown why this virus was able to spread so rapidly and displace the previously circulating noroviruses; however, a number of conserved point mutations were detected both in the RdRp and capsid encoding genes, and most notably, a 3 nucleotide (nt) insertion was seen within the ORF2, localised within the P2 domain. Similarly, another GII-4 variant strain was associated with an increase in out-of-season infection followed by an epidemic season in 2006 [20].

In this paper we describe the amino acid (aa) variation observed in the P2 domain of a number of isolates of GII-4 noroviruses collected between 1997 and 2006, and which represent GII-4 variant strains circulating over a 9 year period in the UK. The strains include two epidemiologically significant strains that gave origin to large epidemics and to out-of-season activity. Potentially immunologically significant variant-specific epitopes in the P2 domain of the capsid protein VP1 are identified and described through homology modelling.

## Methods

### Clinical Samples

Faecal samples were selected from outbreaks occurring between 1997–2006, that had previously been characterised by PCR as being caused by GII-4 norovirus strains, which are archived at the Enteric Virus Unit, Centre for Infections, Health Protection Agency, London. Samples were prepared as 10% suspensions in balanced salt solution (Medium 199, Sigma, Dorset, UK) prior to nucleic acid extraction.

### Nucleic Acid Extraction

A total of 250 µl of the 10% sample suspensions was used for nucleic acid extraction using the guanidium isothiocyanate/silica method as previously described [21].

### Reverse Transcription

Extracted nucleic acid was incubated in the presence of 50 pmol of poly(T) primer, tris-HCl buffer at pH 8.3, 5 mM MgCl_2_, 1 mM each dNTP and 200U SuperScript III Reverse Transcriptase (Invitrogen, Paisley, UK). The reverse transcription reaction was performed at 42°C.

### PCR

A region of the ORF2 1521 bp–1524 bp in length and encompassing the N-terminus through to the end of the P2 domain was amplified using primers ORF1/2-F1 (sense primer, nt position 5039–5057) [5′-CTGAGCACGTGGGAGGGCG-3′] and P2R2 (antisense primer, nt position 6537–6560) [5-CCTGCACTCAAACAGAACCCTACC-3]. The reaction mix contained 1× reaction buffer (Invitrogen), 1 mM each dNTP, 2.0 mM MgCl_2_, 20 pmol of each primer and recombinant Taq polymerase (5U) (Invitrogen). The thermal cycling conditions used were: 1 cycle of 94°C, 2′, followed by 30 cycles of 94°C, 45″/60°C, 45″/72°C, 1′ 40″, and a final extension at 72°C, 10′.

### Amplicon Purification

Amplicons were purified either from solution using Montage® PCR Filter Units (Millipore, Watford, UK), or from agarose gels using Geneclean® Spin Kit (Qbiogene, Cambridge, UK), both were performed according to manufacturers' instructions.

### Amplicon Sequencing

The purified amplicons were used as a template in sequencing reactions. In order to sequence the S domain the following primers were used: ORF1/2-F1 (see above), SpeI-F (sense primer, nt position 5068–5095) [5′-AGCTCACTAGTCTGTGAATGAAGATGGC-3′] or Mon381 [22]. Primers used for sequencing of the P2 domain were: P2F (sense primer, nt position 5873–5894) [5′-TGGCAGRTGYACGACTGATGG-3′] or P2R2 (see above). In each sequencing PCR, 10 pmol of primer were used, and 100 fmol of template DNA. All sequencing was performed using the GenomeLab DTCS – Quick Start Kit (Beckman Coulter, High Wycombe, UK) according to the manufacturer's instructions, and a CEQ8000 automated sequencer (Beckman Coulter).

### Sequence Analysis

AA sequence data was deduced from nt data using BioEdit [23], and were analysed using BioNumerics v3.5 (Applied Maths, Kortrijk, Belgium). Here, analysis is focused on the P2 domain (aa position 279–406) and P2 domain clusters (P2x: where x can be the cluster designation) are assigned based on aa positions 296–298 (site A) and 393–395 (site B) as described in Table 1. Clusters were defined by change of one or more aa residues within site A and/or site B.

**Table 1:** Distribution of P2 domain clusters characterised by changes of one or more residue within the motifs defined as site A and site B, each composed of 3 aa positions

Motif		Year										P2 Domain Cluster	Epidemiological Variant	Number of Strains
Site A	Site B	1997	1998	1999	2000	2001	2002	2003	2004	2005	2006			
SHD	N-N	**1**	**10**	**3**								P2a	pre-2002	14
SHD	S-N			**1**			**2**					P2b		3
THD	NSN					**1**	**1**					P2c	2002 epidemic through to 2006	2
THN	NGT						**2**	**4**				P2d		6
TRT	STA								**1**			P2e		1
TRT	SST									**1**	**1**	P2f		2
SRN	STT										**1**	P2g		1
TQN	STT								**10**	**4**	**2**	P2h		16
TQN	NTT									**9**		P2i		9
TQE	STT										**9**	P2j	2006 epidemic	9
TQE	NTT										**1**	P2k		1
TQE	SAT										**1**	P2l		1
TQH	STT										**1**	P2m		1
													*n* =	66

Numbers indicate the number of sequences associated with each P2 domain cluster in each year. Each sequence represents a different outbreak.

### Protein Modelling

The software packages YASARA (www.yasara.org) and Deep View Swiss-PDB Viewer (www.expasy.org/spdbv) were used to model changes in the P2 domain 3D structure. The previously published crystal structure of norovirus strain VA387 was used in the analysis and is available as a PDB file from www.rcsb.org, DOI 10.2210/pdb2obs/pdb [24].

## Results

**Figure 1:**
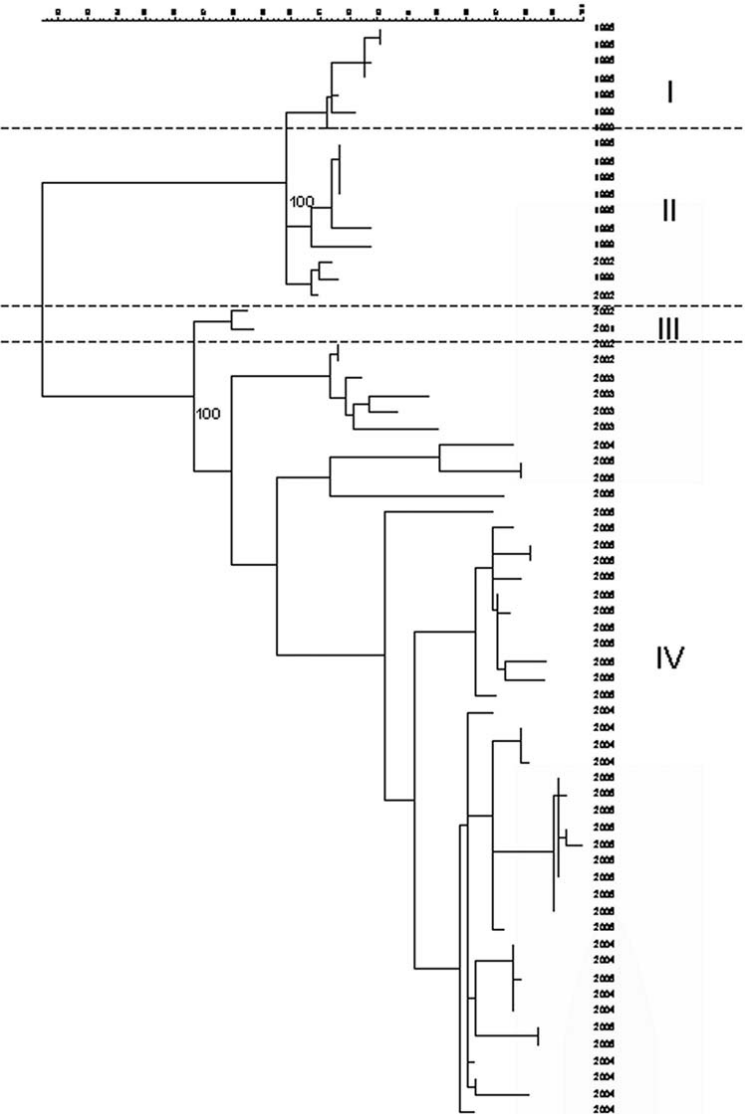
Phylogenetic tree based on nt data showing 66 GII-4 norovirus sequences used in this study. Values indicate significant bootstrap values (1000 pseudoreplicates) that define lineages. Genetic lineages marked on the right (I–IV). Dates indicate the year of isolation

Nucleotide data for 66 P2 domain sequences revealed significant diversity of up to 20% across this region. Analysis of the sequences by the neighbour joining method followed by bootstrap analysis (1000 pseudoreplicates) revealed four genetic lineages defined by bootstrap values ≥99%, Figure 1.

**Figure 2:**
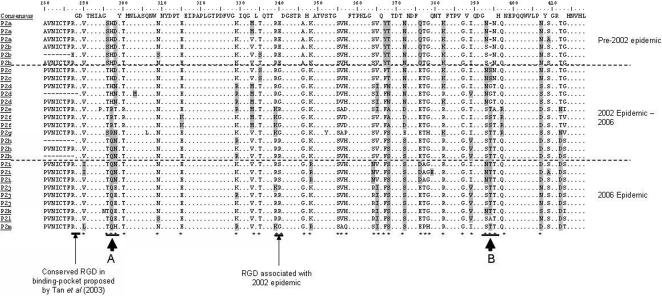
Clustal W alignment of amino acid sequences representing each P2 domain cluster. Deduced amino acid data for 27 of the 66 strains analysed were aligned to represent the 13 P2 domain clusters. Actual numbers of sequences found at each P2 domain cluster are shown in Table 1. * indicates a hotspot (≥2aa changes at the site), and labelled arrows indicate sites putatively associated with antigenic change and cluster transition.

**Figure 3:**
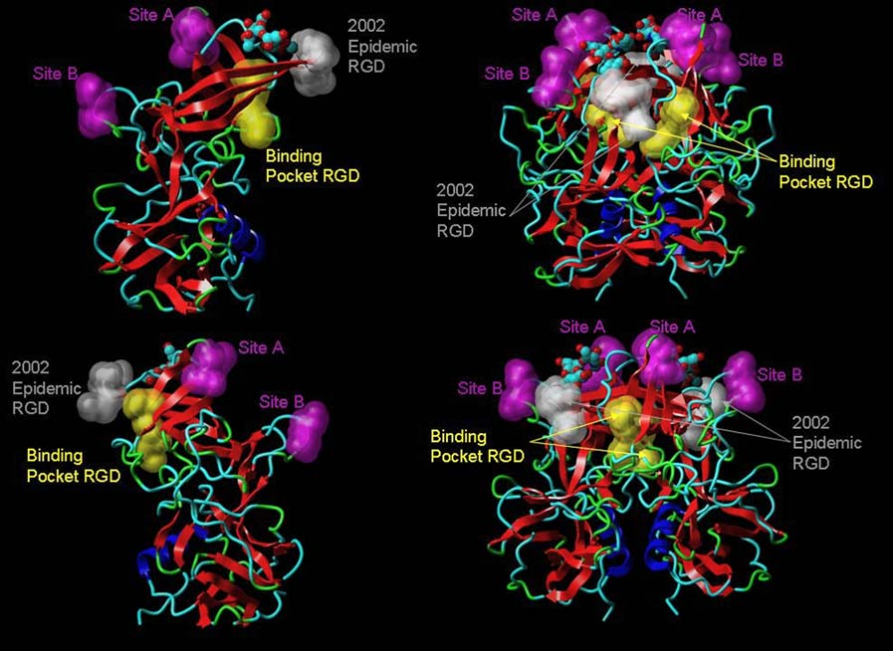
Conformational P domain (strain VA387) showing positions of potentially significant variation hotspots. Hotspots identified as significant amino acid changes were mapped onto the crystal structure of strain VA387. Sites A and B are coloured magenta. RGD motif associated with the binding pocket described by Tan *et al* [25] is coloured yellow. The RGD motif associated with the epidemic in 2002 described in this paper is coloured gray. The sugar molecule indicates the position of the putative binding site proposed by Cao *et al* [24].

Alignment of derived aa sequences revealed 31 hotspots of variation across the P2 domain (aa positions where ≥2aa changes between strains were observed), as well as random point mutations (Figure 2). Hotspots which resulted in homologous aa substitutions and were located in an unexposed region of the capsid structure were deemed unlikely to have a major impact on the antigenic properties of the viruses. Homologous and/or non-homologous changes at aa hotspots that coincided with the epidemic waves and were located in exposed regions of the capsid structure were predicted to have a significant impact on the antigenic properties of the virus. Six positions in which significant aa substitutions that would impact on the biochemical properties and structure of the P2 domain of the virus were identified: aa positions 296–298 (site A) and 393–395 (site B) (Figures 2 and 3). All 6 of these positions localise to external points in the 3D structure of the capsid, and are parts of exposed loops. The aa sequence changes observed at these 6 positions were then correlated with the temporal appearance of epidemiologically significant strains. This analysis identified 13 clusters (P2a-P2m) based on the aa sequence changes at these six positions (Table 1). The biochemically significant changes observed at aa sites 296–298 and 393–395 showed a strong association with the emergence of epidemiologically significant strains.

In addition to the six aa positions identified as sites A and B, a seventh position, aa 340, was noted to be of interest. This position is surface-exposed and non-homologous changes are observed over the time period assessed here. Whilst the aa changes at position 340 do not consistently coincide with the epidemic waves as strongly as those at sites A and B, there is one change that is noteworthy. Synchronised with the 2002 epidemic, an aa substitution occurs at potion 340 which generates an integrin-binding RGD motif, sterically close to both the binding pocket proposed by Tan *et al* [25] and the histo-blood group antigen (HBGA)-binding site identified by Cao *et al* [24]. This motif was detected from 2002–2003, but only in GII-4 strains and not again afterwards.

In order to understand the impact of the cluster-defining aa substitutions observed at sites A and B, three sequences, one representing strains co-circulating before the 2002 epidemic (P2 domain cluster a [P2a]), a second representing the 2002 epidemic strain (P2e), and a third representing the 2006 epidemic strain (P2j) were chosen for homology modelling using as a template the recently described P domain crystal structure of the GII-4 norovirus strain VA387 [24].

**Figure 4:**
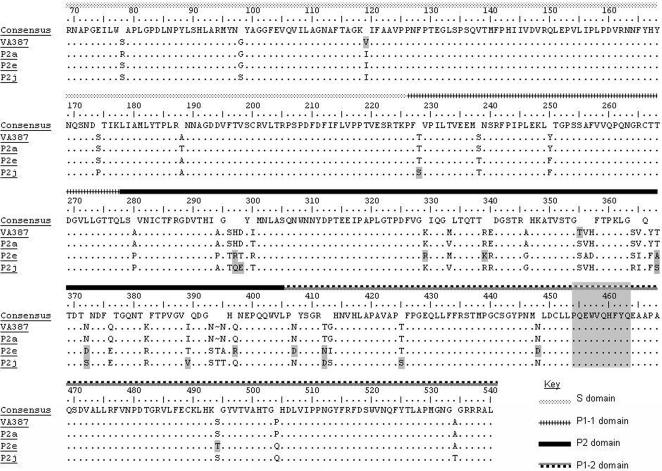
Alignment comparing VA387 strain used in homology modelling with sequences representing epidemiologically significant clusters. Deduced amino acid sequences representing P2 domain clusters P2a, P2e and P2j were aligned with strain VA387 which was used in homology modelling. Key in the figure indicates domains of the VP1 protein. Shaded box indicates the conserved α-helix involved in dimerisation.

Firstly, an aa alignment between P2a, P2e, P2j and VA387 was made (Figure 4). All four sequences shared a high degree of similarity, and strains VA387 and P2a were found to be most closely related as both strains were co-temporal and both lacked the insertion at position 393. Regions of the P1 domain involved in dimerisation (aa positions 454–463) were found to be conserved in the alignment with VA387, and the variable regions identified in the P2 domain showed consistent variation when VA387 was aligned with samples representing clusters P2a, P2e and P2j (Figure 4).

**Figure 5:**
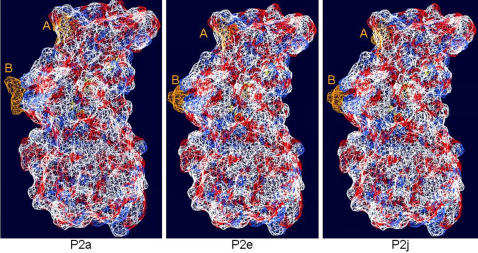
Homology model showing changes in molecular surface. Changes at site A and B (orange) were modelled onto the crystal structure of VA387, and the molecular surface calculated using default settings in Deep View Swiss PDB Viewer program. Significant changes in the shape of the molecular surface were observed at site B between P2 domain clusters P2a and P2e, which was maintained between clusters P2e and P2j. Site A changed more significantly between clusters P2e and P2j than between P2a and P2e.

**Figure 6:**
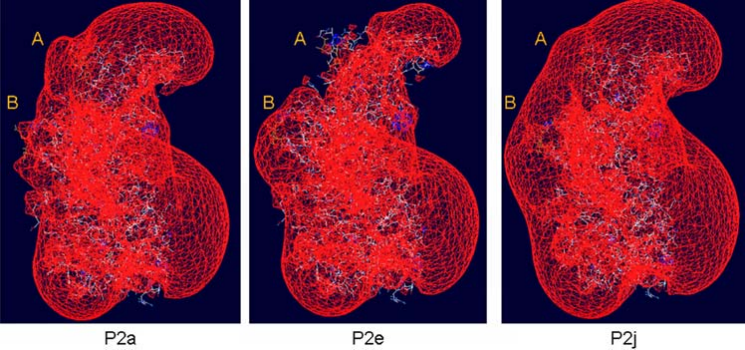
Homology Model showing changes in electrostatic surface. Changes at site A and B were modelled onto the crystal structure of VA387 and the electrostatic surface calculated using default settings in Deep View Swiss PDB Viewer. Significant changes in the electrostatic surface were observed between clusters P2a and P2e, but not between P2a and P2j.

The aa changes at sites A and B of P2a, P2e and P2j were in turn modelled onto the crystalline structure of strain VA387 (Figures 5 and 6). Molecular surface modelling at aa sites A and B revealed substantial changes in the surface shape and surface area of VP1 at both positions. The most significant change between P2a and P2e was the change in surface shape at site B, which was maintained between P2e and P2j, whereas site A appeared to vary substantially in shape between all three clusters (Figure 5). Analysis of the electrostatic surface (Figure 6) showed that at site A the surface was greater for P2a when compared with P2e. Therefore, major changes both in structure and electrostatic charges were detected between P2a (pre-2002) and P2e (2002), and P2e (2002) and P2j (2006), but less pronounced between P2a (pre-2002) and P2j (2006) (Figures 5 and 6). Both molecular and electrostatic surfaces maintained a constant structure in all three clusters in regions of the P domain associated with homodimerisation.

## Discussion

RNA viruses show a high degree of genetic variability and the pool of variants results in a mechanism of virus evolution and survival [26]. Variation at the genome level can result in silent mutations, mutations that are expressed as homologous aa substitutions, or as aa substitutions that change the properties of virus binding or the immunogenicity of virus epitopes and lead to the generation of virus strains with altered tropism or antibody-escape mutants, respectively.

Previous studies have analysed the diversity among GII-4 noroviruses through sequence analysis across the S domain, and shown that the accumulation of point mutations leads to the emergence of variant strains which at times co-circulate, but periodically a variant is selected and is associated with widespread epidemics and out-of-season activity [8], [18], [19]. It has been postulated that the success of these variants was due to either immune evasion or changes in receptor binding. As discussed, this is unlikely to be associated with changes in the S domain as this region is one of the most conserved regions of the norovirus capsid and it is not exposed and is therefore unlikely to contain immunologically relevant epitopes. The high mutation rate and exposed structure of the P2 domain of the capsid protein has led to the assumption that receptor binding sites and key antigenic determinants are localised in this region [24], [25]. In this paper we assess the diversity of GII-4 norovirus strains at the hypervariable P2 domain suspected to be responsible for virus attachment and immune reactivity, at the nucleotide sequence level, deduced aa level and homology analysis through comparison with the crystal structure of a GII-4 P domain, and related these changes to the emergence of epidemic strains.

The analysis presented in this paper showed a high level of diversity in the P2 domain, far greater than that identified in the S domain, and the identification of numerous hotspots for mutation (Figure 2). Detailed analysis of the substitutions at these hotspots and their mapping onto the crystal structure of VA387 revealed two sites, both three aa residues in length, one located near the N-terminus and one near the and C-terminus of the P2 domain (Figure 2 and Table 1). Both sites were located in exposed loops of the P2 domain, and may play an important role in immune responses. These two sites changed in a temporal fashion and significant changes at these sites coincided with the appearance of epidemiologically significant virus strains in the population which resulted in epidemics of gastroenteritis. In addition, it was observed that site A is in close proximity to the HBGA-binding site in the 3D structure of the P domain of the capsid [24]. Conformational changes at this site may also have an impact on binding of these viruses to host cells.

Random neutral drift of aa sequences in proteins has long been established [27], and mapping of such changes has been described using neutral networks [28]. A neutral network can be regarded as a group of genotypes that are linked by point mutations which are selectively neutral. This results in grouping of virus strains by epitope structure rather than sequence diversity.

**Figure 7:**
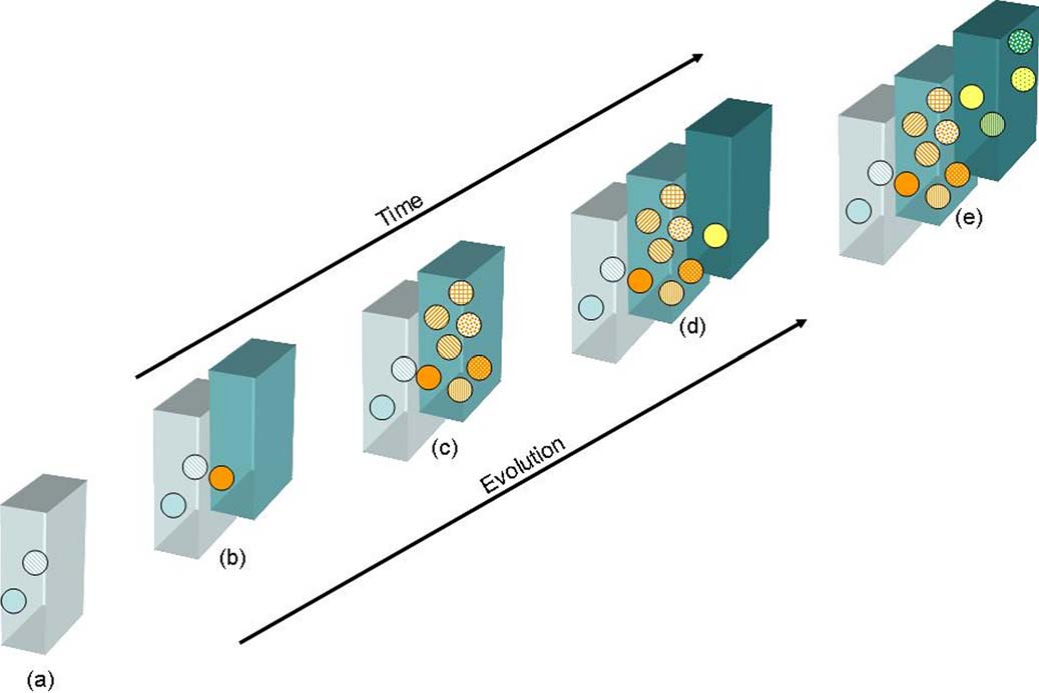
Representation of genotype-to-phenotype mapping of the drift of virus clusters through connected neutral networks. Each coloured box represents a neutral network, where although a number of different amino acid sequences exist in the sequence space, the resulting conformational protein is homologous within each network, but distinct from every other network. (a) Two clusters (blue dots) were identified comprising sequences pre-2002 epidemic (Table 1), and share epitope shape at sites A and B, although differ in primary amino acid sequence. (b) Acquisition of point mutations at sites A and B around 2001–2002 that are sufficient to alter the conformational structure of VP1 occur concurrently, and thus this virus (orange dot) moves from one neutral network to occupy sequence space on a different neutral network. Change in conformational structure of putative epitope sites A and B following transition to a different neutral network gives these viruses a selective advantage as they are able to evade existing immunity in the population, thus resulting in the epidemic of gastroenteritis observed in 2002, as antibody-driven selection among the virus populations favours those on this, different, neutral network. (c) Following selection, the virus drifts through the sequence space of the neutral network, acquiring homologous substitutions in the primary amino acid sequence. (d) Acquisition of point mutations around 2005–2006 that connect the virus to a third neutral network then puts this virus at a selective advantage as it can evade existing immunity. Again, the result in epidemic gastroenteritis as observed in 2006 as selection drives transition from one neutral network to another. (e) The virus now drifts neutrally through the sequence space of this neutral network.

The aa sites A and B showed both significant and neutral aa changes. Periodically, significant aa substitutions occurred concomitantly at aa sites A and B, and this resulted in both cluster transition (progression from one defined cluster to another through aa changes at site A and/or site B) and resulting in the appearance of epidemiologically significant virus strains (Table 1). In this context, GII-4 norovirus strains circulating before 2002 constituted a neutral network of viruses which were antigenically indistinguishable from each other, although there was significant sequence diversity among them. Mutations, as proposed in this paper, at sites A and B resulted in conformational changes that altered the antigenic properties of the viruses, and distinguished them from those circulating in previous years, leading to the emergence of a major epidemic [18] possibly due to lack of herd immunity. Genetic drift continued to generate mutations and increase diversity among the strains circulating during and after this epidemic constituting another neutral network of viruses which although diverse at the nt and aa level, were antigenically equivalent, until significant changes occurred again at sites A and B which coincided with another epidemic wave that lead subsequently to a third neutral network (Figure 7). It has recently been shown that the characteristic punctuated appearance of antigenically novel strains of influenza A virus can be described by neutral networks [29] in a similar way to that described for GII-4 noroviruses here.

Homology modelling using the recently published crystal structure of GII-4 strain VA387 [24] revealed structural data which supported our conclusion that sites A and B together act as variant-specific epitopes. Both sites localised to surface exposed loop structures (Figure 3), and at both sites aa mutations associated with cluster transitions were found to also be linked to substantial variation in the molecular and electrostatic surface of the protein (Figure 5 and Figure 6). The conformational changes observed at aa positions 297, 298, 394 and 395 coincided with the appearance of an epidemiologically significant variant and antigenic cluster transition (Table 1 and Table 2). Changes at these positions create a substantial change in bulk and biochemical properties among the residues at their respective site (Table 2). These structural and electrostatic changes may be sufficiently prominent to obscure the site from recognition by existing antibodies (Figure 5 and Figure 6), thus permitting the virus to escape the host pre-existing immunity.

**Table 2:** Key mutations in sites A and B

			Cluster		
			P2a	P2e	P2j
**Site A**	**297**	**Amino Acid**	H	R	Q
		**Properties**	Basic	Basic	**Polar**
		**Size Change**		**Increase**	**Decrease**
	**298**	**Amino Acid**	D	T	E
		**Properties**	Acid	**Polar**	**Acid**
		**Size Change**		**Increase**	**Decrease**
**Site B**	**394**	**Amino Acid**	-	T	T
		**Properties**		**Polar**	Polar
		**Size Change**		**Increase**	None
	**395**	**Amino Acid**	N	A	T
		**Properties**	**Polar**	**Neutral**	**Polar**
		**Size Change**		**Decrease**	**Increase**

Characteristics in **bold text** indicate a change from previous cluster, i.e. P2a → P2e, or, P2e → P2j. ‘-’ indicates no residue present, and was the site of the subsequent 3nt insertion.

A study similar to that presented here, looking at the sequence diversity among GII-4 noroviruses and *in silico* analysis of the changes observed, was published recently by Siebenga *et al* [9], however the interpretation of the data by Siebenga *et al* is not in agreement with the data presented here. The study of Siebenga *et al* concludes that five individual aa positions spread across the entire P domain displayed consistent changes which were informative for distinguishing new epidemic variants [9]. In contrast, we predict that only sites within the P2 domain that are exposed in the conformational protein will affect the immunogenicity of the virus. There are a number of significant differences between the two studies which may have resulted in the conflicting interpretations of essentially equivalent data sets. Firstly, Siebenga *et al* choose to model the aa changes they have observed onto the crystal structure of Norwalk virus [4], whereas our homology modelling study was performed using the recently described crystal structure for the virus VA387 [24]. Norwalk virus is a GI virus and has a shorter capsid protein, including a shorter P2 domain, whereas VA387 is a GII-4 norovirus, and therefore VA387 is an accurate model for the sequence data used in analyses involving GII-4 strains. Secondly, the association of the sequence data with epidemiological information is an important part of the analysis in both papers. In this paper, we show that the changes at 6 specific aa positions occur by a process of drift and that significant changes at these positions coincide with epidemics observed across Europe in 2002 [18] and 2006 [J Harris, personal communication; 20], and furthermore, the trend is observed continuously for all isolates from all years analysed. In contrast, the study of Siebenga *et al* identifies 2004 as an epidemic year, and although equivalent variant strains were circulating in the UK in 2004, such an epidemic was not observed in the UK or throughout Europe [J Harris, personal communication]. Furthermore, their analysis specifically excludes sequence data from 2006 epidemic strains. Finally, Siebenga *et al* base their conclusions on sequence analysis and homology modelling alone, however details of the effects the changes at the 5 positions identified in their paper would have on the conformation of VP1 are not addressed in detail. In this study, we introduce associations between the reported epidemiology of noroviruses, the sequence diversity observed among temporally separated viruses, and link this with these biochemical properties of the capsid protein.

This analysis presented in this paper has predicted sites within the P2 domain that may be immunologically significant but it remains to be proven that mutation at these sites result in antibody-escape mutants. Following the *in silico* identification of putative epitopes here, work is currently underway to assess the functionality of these sites *in vitro*.
